# 

**Published:** 1874-03

**Authors:** 


Alumni of the Chicago Medical College.—The members of the Alumni Association of the Chicago Medical College will hold their annual meeting at the college, on Tuesday, March 10th, at 10 o'clock, A.M.

